# Gastrointestinal Hemorrhage Due to Splenic Artery Aneurysm Pancreatic Duct Fistula in Chronic Pancreatitis

**DOI:** 10.1155/1993/81291

**Published:** 1993

**Authors:** Christian Seiler, Leslie H. Blumgart

**Affiliations:** 1 Department of Visceral and Transplantation Surgery University of Berne Switzerland; 2 Sloan Memorial Cancer Center New York USA

## Abstract

Gastrointestinal hemorrhage due to splenic artery aneurysm pancreatic duct fistula in chronic pancreatitis is rare. It is, however, important to diagnose this condition particularly in patients having chronic pancreatitis, since it may result in a life-threatening situation. The diagnosis is usually difficult to establish and it may take repeated admissions for intermittent gastrointestinal bleeding until the real source is recognized. Clinical attacks of epigastric pain followed by GI-bleeding 30–40 minutes later are characteristic. Occasionally these attacks are followed by transient jaundice. The present case report describes this rare complication and reviews the current literature.


*HPB Surgery*, 1993, Vol. 7, pp. 149–155
Reprints available directly from the publisher
Photocopying permitted by license only

© 1993 Harwood Academic Publishers GmbH
Printed in the United States of America

### CASE REPORT

### GASTROINTESTINAL HEMORRHAGE DUE TO SPLENIC ARTERY ANEURYSM PANCREATIC DUCT FISTULA IN CHRONIC PANCREATITIS

### A case report and review of the literature

CHRISTIAN SEILER and LESLIE H. BLUMGART*
*Department of Visceral and Transplantation Surgery, University of Berne and Sloan Memorial Cancer Center, New York, USA**

*(Received 10 September 1992)*

Gastrointestinal hemorrhage due to splenic artery aneurysm pancreatic duct fistula in chronic pancreatitis is rare. It is, however, important to diagnose this condition particularly in patients having chronic pancreatitis, since it may result in a life-threatening situation. The diagnosis is usually difficult to establish and it may take repeated admissions for intermittent gastrointestinal bleeding until the real source is recognized. Clinical attacks of epigastric pain followed by GI-bleeding 30–40 minutes later are characteristic. Occasionally these attacks are followed by transient jaundice. The present case report describes this rare complication and reviews the current literature.

### INTRODUCTION

While hemobilia is now well recognised (Sandblom), hemorrhage through the pancreatic duct, termed "hemowirsungia" by Bismuth[1], "hemosuccus pancreaticus" by Sandblom[2], "hemoductal pancreatitis"[3] and"wirsungorrhagia"[4] is less frequently documented as a cause of gastrointestinal hemorrhage.

While close to 110 cases of bleeding through the pancreatic duct have been published[5–13] only 26 cases due to rupture of a splenic artery aneurysm into the pancreatic duct in combination with pancreatitis are to be found in the literature[14–18].

The typical triade of attacks of gastrointestinal hemorrhage, pain in the epigastric area and occasional jaundice should blood enter into the biliary tract, are similar to the symptomatology of hemobilia and while undiagnosed upper gastrointestinal bleeding in the presence of chronic pancreatititis should suggest the diagnosis, the

Address correspondence to: Christian Seiler, Department of Visceral and Transplantation Surgery, University of Berne, Switzerland condition may persist for long periods of time and be the true cause of repeated gastrointestinal hemorrhage remaining masked.

This paper reports the case of gastrointestinal hemorrhage due to splenic artery aneurysm pancreatic duct fistula in chronic pancreatitis. The relevant literature is reviewed.

### CASE REPORT

A 61 year old male was admitted to hospital in 1990 having had several attacks of upper abdominal pain followed by the passage of bright red blood per rectum, occurring some 30 minutes after the onset of each attack. The history revealed that the patient had had a ski accident with chest trauma in 1970. Multiple left sided rip fractures and an injury to the right shoulder were treated at that time but later in the same year he began to suffer from recurrent attacks of acute pancreatitis. Ten years later he developed a sudden onset of abdominal pain followed shortly afterwards by melaena. At that time no source of bleeding was detected at gastroscopy or colonoscopy. The history was otherwise unremarkable.

On admission to hospital after another attack of abdominal pain it was found that the hemoglobin was 7.8 g/% but other hematological investigations including liver function tests and pancreatic enzymes were normal. Colonoscopy was unremarkable and gastroscopy revealed several non bleeding gastric varices. A CT scan of

**Figure 1** Angiography of celiac trunk. Arrow shows "aneurysma-like" dilatation of splenic artery. (*See colour plate at the back of this issue*).

the abdomen detected multiple calcifications in the head of the pancreas and Duplex sonography showed thrombosis of the splenic vein and splenomegaly. Blood transfusion was necessary and the patient was then transferred to the Department of Visceral Surgery at Inselspital Bern with a diagnosis of portal hypertension associated with splenic venous thrombosis. Examination of the patient was normal except for gross splenomegaly.

While in hospital the patient suffered a further attack of melaena associated with upper abdominal pain and shock. Gastroscopy again showed non bleeding gastric fundal varices and emergency colonoscopy no source of bleeding but blood in the entire length of the colon. A repeat colonoscopy after bowel preparation was normal. Emergency coeliac access and superior mesenteric angiography did not

**Figure 2** ERCP showing a stenosis of the pancreatic duct in the neck of the pancreas in projection on the spine. (*See colour plate at the back of this issue*).

reveal any source of bleeding. However an aneurysm of the mid portion of the splenic artery was detected (Figure 1). Radio-labeled erythrocyte scintigraphic scanning proved negative. Endoscopic retrograde cholangiopancreatography (ERCP) (Figure 2) showed a stenosis of pancreatic duct in the region of the neck, very suggestive of post traumatic change combined with multiple ductal irregularities and evidence of distal pancreatitis in the tail of the pancreas.

The patient was taken to operation and at laparotomy after opening the lesser sack a grossly scared neck of the pancreas was identified lying directly over the vertebral column. A posttraumatic aneurysm of the splenic artery 5 mm in diameter precisely within the area of scaring was also evident. Thrombosis of the splenic vein was confirmed. Splenectomy and pancreatic tail resection including the entire aneurysm was performed. Examination of the specimen revealed splenic artery aneurysm-pancreatic duct fistula in a chronically inflammed pancreas (Figure 3 showing the fistula macroscopically). The patient had an uneventful postoperative course and was well at follow up one year later.

### DISCUSSION

Chronic pancreatitis complicated by splenic artery aneurysmal rupture into the pancreatic duct with gastrointestinal hemorrhage is rare but life-threatening. The key to therapy is the high diagnostic index of suspicion in patients with chronic pancreatitis who present with gastrointestinal bleeding of unknown cause. A review of the literature reveals that every patient suffering from the condition has

**Figure 3** Splenic artery with aneurysm (arrow). *(See colour plate at the back of this issue).*

complained of intermittent epigastric pain followed by episodes of acute gastrointestinal bleeding which occur some 30 to 40 minutes after the onset of symptoms. Characteristically multiple admissions to hospital precede the correct diagnosis. Indeed 70% of patients required more than one admission to hospital prior to diagnosis and indeed symptoms may have been present for up to 10 years (Table 1)[14–18]. Angiography has revealed the existence of a splenic artery aneurysm in 25 of

**Table 1** Summary of 27 reported cases (including present case)

| | |
|---|---|
| *Number of Admissions* | |
| Mean | 2.7 |
| Range | 1–3 |
| *Duration of Symptoms Until Diagnosis (years)* | |
| Mean | 2.44 |
| Range | 1–120 mo. |
| Age (years) | |
| Mean | 54.3 |
| Range | 16–80 |
| Male:Female | 1.7:1 |
| Angiography | |
| Splenic Artery Aneurysm | 25 |
| Leak into pancreatic duct | 5 |

**Figure 4** Pancreatic duct (arrow). Fistula from splenic artery aneurysm into the pancreatic duct. Head of the probe (arrow). *(See colour plate at the back of this issue)*

27 cases (including the present case). In two cases the diagnosis was found at autopsy. A frank communication with a leak of contrast into the pancreatic duct was demonstrated in only 5 patients. Emergency gastrointestinal endoscopy only rarely identifies bleeding from the papilla of Vater since the bleeding is intermittent and is usually stopped by the time examination is carried out or visibility may be impaired by active hemorrhage. ERCP usually reveals the ductal irregularities characteristic of pancreatitis but not the aneurysm.

Treatment may be by embolisation (Hemingway, Allison, Blumgart) or by surgical operation. Of the 27 reported cases operation was carried out in 26. The standard surgical procedure being pancreatectomy, aneurysectomy and splenectomy. There have been no operative deaths reported in the literature and short term follow up was uneventful in each case. The one patient treated by radiological embolisation also fared well. Indeed while operation will continue to be the first choice for many the availability of good interventional radiology should encourage embolisation in suitable cases and it may even by used in the unstable patient to bide time for resuscitation and subsequent surgery[20].

The key to successful treatment rests on a high index of diagnostics suspicion, good angiographic studies and a surgical procedure or interventional radiological approach, which effectively occludes or removes the aneurysm and thus the risk of bleeding. At surgical operation it is usually necessary to exise the aneurysm along with the damaged pancreatic tail and spleen.

### *References*

1. Bismuth, H.M., Charton, B., Martin, E., Hernandes, C. and Hepp, J. (1970) Les Hémorragies digestives provenant des canaux pancréatiques. *(Les Hémowirsunguies) Seance*, **12**, Octobre, p. 735
2. Sandblom, Ph. (1970) Gastrointestinal hemorrhage through the pancreatic duct. *Annals of Surgery*, 61–66
3. Longmire, W.P. and Rose, III A. (1973) Hemoductal pancreatitis. *Surgery, Gynecology & Obstetrics*, **136**, 246–250
4. Vankemmel, M., Paris, J.C., L'Hermine, C. and Houcke, M. (1973) Les Wirsungorragies. *Médecine Chirurgie Digestive*, **5**, 83–91
5. Marre, Ph., Place, G., Botreau, Y., Masbou, P. and Darnault, P. (1984) Wirsungorragie par rupture d'un volumineaux anévrysme athéromateux de l'artère hépatique. *Chirurgie*, **110**, 655–661
6. Le Neel, J.C., Leborgne, J., Heloury, Y., Deret, Ch., Lavignolle, A. and Malvy, P. (1984) Wirsungorragies des pancréatites chroniques. *Journal de Chirurgie*, **11**, 121
7. Van Rooyen, W., Van Blankenstein, M., Eeftnick-Schattenkerk, E.M., De Vries, J.E., Obertop, H., Bruining, H.A. and Van Houten, H. (1984) Haemorrhage from the pancreatic duct: a rare form of upper gastrointestinal bleeding. *British Journal of Surgery*, **71**, 137–140
8. Haller, J., Pena, C. and Dargan, E. (1966) Massive upper gastrointestinal hemorrhage due to pancreatitis. *Archives of Surgery*, **93**, 567–572
9. Koehler, P.R., Nelson, J.A. and Berenson, M.M. (1976) Massive extraenteric gastrointestinal bleeding: Angiographic diagnosis. *Radiology*, **11**, 41–44
10. Melville, G.E. and Maxwell, D.D. (1983) Massive lower GI hemorrhage in hemoductal pancreatitis. *Gastrointestinal Radiology*, **8**, 145–146
11. Yoshikai, T., Murakami, J., Nishihara, H. and Oshiumi, Y. (1989) Hemosuccus pancreaticus: CT manifestations. *Journal of computer assisted tomography*. **10**(3), 510–512
12. Lerebours, E., Teniere, P. and Janvresse, A. (1984) Traitement par embolisation artérielle d'un cas de wirsungorragie. *Journale de Chirurgie*, **121**(5), 339–341
13. Salmassi, S. (1983) Pancreatica magna aneurysm: Rupture into the pancreatic duct. *Southern Medical Journal*, **76**, 1565–1567
14. Harper, P.C., Gamelli, R.L. and Kaye, M.D. (1984) Recurrent hemorrhage into the pancreatic duct from a splenic artery aneurysm. *Gastroenterology*, **87**, 417–420

15. Corbeau, A., Sahel, J., Fraissinet, R., Courjaret, P., Burelle, H. and Clément, J.P. (1978) Wirsungorragie, diagnostic artériographique en urgence. *Journal of Radiology Electrol.*, **59**, 275–278
16. Yokoyama, I., Hashmi, M.A., Srinivas, D., Shaikh, K.A., Levine, S.M., Sorokin, J.J. and Camishion, R.C. (1984) Wirsungorrhagia or hemoductal pancreatitis: Report of a case and review of the literature. *American Journal of Gastroenterology*, **79**, 764–768
17. Arisi, C., Colombo, L., Arisi, L. and Alessiani, M. (1986) Le emorragie attravesso il Wirsung. *Minerva Chirurgica*, **41**, 1945--1955
18. Uomo, G., Visconti, M., Ziviello, M., Galloro, G., Rabitti, P. and Galloro, V. (1989) Emorragia nel dotto di Wirsung da aneurismi dell'arteria splenica in corso di pancreatite cronica. *Minerva dietologica e gastroenterologica*, **35**, 55–60
19. Steckman, M., Dooley, M., Jaques, P. and Powell, D. (1984) Major gastrointestinal hemorrhage from peripancreatic blood vessels in pancreatitis. Treatment by embolotherapy. *Digestive Diseases and Sciences*, **29**(6), 486–497
20. Seiler, Ch., Fielding, G., Triller, J., Schultheiss, H.R. and Blumgart, L.H. (1991) Rectal bleeding associated with chronic pancreatitis. *Hepato-Pancreatic-Biliary Surgery*, **3**, 199–203

*(Accepted by S. Bengmark 10 September 1992)*

